# Molecular hybridization modification improves the stability and immunomodulatory activity of TP5 peptide

**DOI:** 10.3389/fimmu.2024.1472839

**Published:** 2024-11-11

**Authors:** Junyong Wang, Yuan Tang, Xuelian Zhao, Zetao Ding, Marhaba Ahmat, Dayong Si, Rijun Zhang, Xubiao Wei

**Affiliations:** ^1^ State Key Laboratory of Animal Nutrition and Feeding, College of Animal Science and Technology, China Agricultural University, Beijing, China; ^2^ Institute of Microbiology, Xinjiang Academy of Agricultural Sciences, Xingjian Laboratory of Special Environmental Microbiology, Urumqi, China

**Keywords:** hybrid peptide, physiological stability, immunomodulatory activity, TLR2, molecular dynamics simulations, cytokines

## Abstract

Thymopentin (TP5) plays an important role in host immunomodulation, yet its bioavailability is significantly limited by its short half-life. YW12D is a peptide with strong stability but relatively weak immunoactivity. Tuning the physicochemical properties of such molecules may yield synthetic molecules displaying optimal stability, safety and enhanced immunological activity. Here, natural peptides were modified to improve their activity by hybridization strategies. A hybrid peptide YW12D-TP5 (YTP) that combines TP5 and YW12D is designed. The half-life of YTP in plasma is significantly longer than that of YW12D and TP5. YTP also displays an improved ability to protect the host from CTX-induced weight loss and thymus and spleen indices decrease than YW12D and TP5. In addition, YTP promotes dendritic cell maturation and increases the expression of cytokines IL-1β, IL-6, TNF-α and immunoglobulins IgA, IgG, and IgM. A combination of antibody-specific blocking assay, SPR, molecular dynamics simulations and western blotting suggest that the immunomodulatory effect of YTP is associated with its activation of the TLR2-NF-кB signaling axis. In sum, we demonstrate that peptide hybridization is an effective strategy for redirecting biological activity to generate novel bioactive molecules with desired properties.

## 1 Introduction

Immune responses are defensive reactions of humans and animals to microorganisms, macromolecules, chemicals and even self-antigens that are recognized as foreign (1–3). As a state of temporary or permanent immune dysfunction, immunosuppression can make an organism more susceptible to infection, organ injury, and cancer due to damage to the immune system (4, 5). Researchers have taken a long time to develop agents to treat immunosuppressive diseases, but progress remains slow. Therefore, it is necessary to explore and develop new immunomodulatory agents to prevent and treat immunosuppressive diseases.

In recent years, peptides have gained a wide range of attention in medicine and biotechnology, and therapeutic peptide research is also currently experiencing prosperity (6–9). Peptides offer a unique strategy for drug design because they elicit minor side effects and offer multiple biological functions and high efficacy (9–13), which would allow them to successfully comply with the stringent standards set for clinical trials. More importantly, peptides are selective and efficacious signaling molecules that bind to specific surface receptors (e.g., Toll-like receptor 2, TLR2) to trigger intracellular effects (14). Pattern recognition receptors, a key class of these molecules, have been extensively studied in this context. The immune system recognizes pathogen-associated molecular patterns and is involved in sensing endogenous danger signals through a series of pattern recognition receptors, especially Toll-like receptors. TLR2, as a key receptor in the TLR family, plays a fundamental role in the development of adaptive immunity and the regulation of immune responses (6, 11, 15, 16). A recent study has indicated that TP5 can exert its immune effects by binding to TLR2 (17). Given their attractive pharmacological profile and intrinsic properties, peptides represent an excellent starting point for the design of novel therapeutics, and their outstanding biochemical properties have been translated into excellent safety, tolerability, and efficacy in humans and animals (18).

Thymopentin (TP5), the Arg32–Tyr36 fragment derived from thymopoietin, restores the thymic atrophy induced by immunosuppressants and regulates immune responses (19, 20). Given its excellent immunomodulatory activity and low cytotoxicity, TP5 has been used clinically to treat patients with a variety of immunodeficiency disorders, such as cancer, hepatitis B virus infection and acquired immunodeficiency syndrome (17, 19, 21, 22). Although TP5 plays a crucial role in immunomodulation, it exhibits a short half-life (12, 23, 24), which severely hampers its clinical development. YW12D is a short amphipathic peptide with strong stability, low cytotoxicity, but relatively weak immunoreactivity (6, 25). In recent years, several approaches have been developed to optimize the design of synthetic peptides with improved biological activities and bioavailability, including computer design, synthetic libraries, template-assisted methodologies, and sequence mutations (26–28). Hybridization, however, has not been as fully exploited, as a route to rational peptide design (10, 29, 30). In the present study, we designed a hybrid peptide, YTP, by combining the peptide YW12D with TP5. The peptide was hypothesized to have a stronger immunoregulatory activity and longer half-life than its parental peptides. We further investigated whether the hybrid peptide could provide more effective therapy against cyclophosphamide (CTX)-induced immunosuppression and elucidated the underlying mechanisms.

## 2 Materials and Methods

### 2.1 Peptide design and synthesis

The hybrid peptide YTP was designed by combining the peptides YW12D and TP5. The complete amino acid sequences of the hybrid peptide YTP and its parental peptide are listed in **Table 1**. The hybrid peptide YTP and its parental peptides YW12D and TP5 were synthesized (95% purity) by KangLong Biochem Ltd. (Jiangsu, China) and their molecular weights were determined by matrix-assisted laser desorption/ionization time-of-flight mass spectrometry (MALDI-TOF-MS). The peptides were stored at -80°C until analysis.

**Table 1:** Design and sequence of parental and hybrid peptides.

Peptides	Sequence
YW12D	YVKLWRMIKFIR
TP5	RKDVY
YTP	YVKLWRMIKFIRRKDVY

### 2.2 Circular dichroismspectroscopy analysis

CD spectroscopy was used to analyze the secondary structure of the hybrid peptide YTP. The peptide was dissolved in sterile water and 25 mM sodium dodecyl sulfate (SDS) at a concentration of 0.1 mg/mL. CD measurements were performed over an ultraviolet (UV) spectral range of 190–250 nm at 25°C using a Jasco J-810 spectropolarimeter.

### 2.3 Cell culture

Murine macrophage cells (RAW264.7) were purchased from the Shanghai Cell Bank, the Institute of Cell Biology, China Academy of Sciences and cultured in Dulbecco’s modified Eagle’s medium (DMEM; HyClone, UT, USA). The DMEM medium was supplemented with 10% (v/v) fetal bovine serum (Bioscience) and 1% (v/v) penicillin/streptomycin (HyClone) and maintained at 37°C in a humidified atmosphere (5% CO_2_, 95% air).

### 2.4 Cell viability assay

RAW264.7 viability was measured using a Cell Counting Kit-8 (CCK-8) Assay Kit (Dojinbo, Japan). Cells were seeded in 96-well plates at a density of 2 × 10^4^ cells/well and treated with or without YW12D, TP5 and YTP peptides (10–80 μg/mL). After incubation for 24 h, the cells were then incubated in CCK-8 reagent for 4 h at 37°C. The absorbance of each well was measured at 450 nm using a 96-well microplate reader. Cell viability was evaluated as follows:


(1) $$Cell\;viability\;\left(\%\right)=\left(OD_{450\;(sample)}/OD_{450\;(control)}\right)\times 100\%$$

### 2.5 Immunomodulatory activity in the RAW264.7 cell line

RAW264.7 cells were stimulated with or without 10 μg/mL peptides for 24 h at 37°C. Levels of tumor necrosis factor (TNF)-α, interleukin (IL)-6 and IL-1β in the cell culture supernatant were qualified using ELISA kits (eBioscience, San Diego, USA) according to the manufacturer’s instructions.

### 2.6 Stability of YTP in rat serum

Rat plasma was collected by centrifuging whole blood from healthy adult rats. YW12D, TP5, and YTP (10 µg/mL) were incubated for different time at 37°C in rat plasma. The samples were collected into prechilled tubes containing 1 mL of acidic acetone (hydrochloric acid/acetone/H_2_O, 1:40:5) and centrifuged at 2, 000 × g for 20 min at 4°C. The obtained precipitates were dried in vacuum. Then, the dried precipitates were dissolved in 0.5 mL of 1 M acetic acid. The peptide analysis was conducted using HPLC. The half-lives of the peptides were calculated by a logarithm-linear regression analysis of the peptide concentrations.

### 2.7 Animal model

Sixty Balb/c female mice (6-8 weeks of age) were purchased from Charles River (Beijing, China) and maintained in cages under specific-pathogen-free (SPF) conditions. Throughout the experimental period, feed and fresh water were provided ad libitum to all mice. All procedures and experiments were performed in accordance with guidelines provided by the Institutional Animal Care and Use Committee of China Agricultural University.

All animals were randomly divided into the following 5 groups (12 mice in each group): control group; CTX group, treated with CTX (80 mg/kg mouse weight); CTX+YW12D group, mice pretreated with CTX followed by 10 mg/kg YW12D treatment; CTX+TP5 group, mice pretreated with CTX followed by 10 mg/kg TP5 treatment; and the CTX+ YTP group, mice pretreated with CTX, followed by 10 mg/kg YTP treatment. CTX was intraperitoneally (i.p.) injected into the CTX, CTX+YW12D, CTX+TP5, and CTX+YTP groups once daily for 3 days. From days 4 to 10, 10 mg/kg mouse weight peptides were i.p. injected into mice (CTX+YW12D, CTX+TP5, and CTX+ YTP) each day. An equal volume of sterile saline (HyClone) was administered i.p. to the control group each day. From day 4 to 10, the CTX group was administered an equal volume of sterile saline as a control. Twenty-four hours after the last dose, the mice were sacrificed, and their blood and tissues were collected. The mouse body weights were recorded at the beginning and end of the experiment. Spleen and thymus indices were calculated as follows:


(2) Index(mg/g)=weight of spleen or thymus/body weight

### 2.8 Flow cytometric analysis of T cell subpopulations in spleen

Spleens were collected, ground, and passed through 40-μm-mesh cell strainers to harvest the single cell suspension. The cells were labeled with peridinin-chlorophyll-protein complex (PerCP)-conjugated anti-mouse CD3^+^, allophycocyanin (APC)-conjugated anti-mouse CD4^+^, and fluorescein isothiocyanate (FITC)-conjugated anti-mouse CD8^+^ (BD Pharmingen, America) for 30 min at 4°C. The T lymphocyte subpopulations were determined by flow cytometry (BD Biosciences, Franklin Lakes, NJ, USA).

### 2.9 Measurement of phenotypic molecule expression

Mouse peripheral blood mononuclear cells (PBMCs) were obtained by Ficoll density gradient centrifugation. Cells were suspended in PBS containing 5% FBS and then incubated with 10% (v/v) normal goat serum for 15 min at 4°C. The cells were labeled with FITC-conjugated antibodies specific for major histocompatibility complex class-II (MHC-II; BD Pharmingen, America). The surface expression of MHC-II was determined by flow cytometry (BD Biosciences, Franklin Lakes, NJ, USA).

### 2.10 Serum cytokine and immunoglobulinmeasurements by ELISA

Mouse whole blood was centrifuged at 1,000 × g for 20 min and serum was collected. Levels of TNF-α, IL-6, IL-1β, IgG, IgA, and IgM in the serum were measured by ELISA (Solarbio, Beijing, China).

### 2.11 Molecular docking

The three-dimensional (3D) structure of the hybrid peptide YTP was built using I-TASSER (https://zhanglab.ccmb.med.umich.edu/I-TASSER/). The constructed 3D structure mode of YTP was next subjected to molecular docking and visualized by PYMOL software. The crystallographic structure of TLR2 was obtained from the Protein Data Bank (PDB ID: 1FYW). For protein-protein docking, Zdock 3.0.2 was employed to acquire the initial complex structure of TLR2-YTP. Altogether, 3600 decoy structures were obtained from the Zdock binding prediction, from which the best decoy structure with the lowest energy was chosen for the following flexible docking analysis. One thousand decoy structures were acquired by flexpepdock (http://flexpepdock.furmanlab.cs.huji.ac.il/), among which the plausible TLR2-YTP docking structure with the lowest binding energy score was selected for analysis.

### 2.12 Molecular dynamics simulations

GROMACS 2020.6 software was used to perform MD simulations of the YTP-TLR2 complex with the AMBER99SB-ILDN force field (31). The complex was placed centrally in a dodecaherdron box with a size of 1.2 nm and dissolved in water (32). Na^+^/Cl^-^ ions were added to keep the system in a neutral environment (32). Energy minimization was performed using the steepest descent algorithm and sustained until the maximum force < 1000 kJ/mol/nm with a step size of 0.01 (33). NVT and NPT ensembles were simulated using the leap-frog algorithm for 1 ns, and the temperature and pressure were set to 310 K and 1 bar, respectively (34). At the end a 200 ns MD simulation was performed. After the stimulation, the trajectory file was analyzed using GROMACS, and the root mean square deviation (RMSD) and radius of gyration (Rg) of the system were obtained.

### 2.13 Specific binding of YTP to TLR2

RAW264.7 cells were treated with PBS or an anti-mouse mAbTLR2 complex (MTS510 Ab) (eBioscience, San Diego, CA, USA), followed by treatment with or without 10 μg/mL YTP peptide and further incubated at 37°C for 24 h. TNF-α, IL-6, and IL-1β levels in the culture supernatant were detected by ELISA.

Surface plasmon response (SPR) experiments were conducted using a ProteOn XPR36 instrument (Bio-Rad, Hercules, CA, USA) with a ProteOn GLH sensor chip (Bio-Rad) at 25°C. The running buffer (PBS with 1% Tween 20) was continuously passed into the reaction chamber at 30 μL/min. The SPR sensing chip with recombinant TLR2 (R&D Systems) was immobilized by amino coupling to capture YTP. The binding affinity of YTP to TLR2 was examined using peptide concentrations of 0, 1.25, 2.5, 5, and 10 mM. The running buffer was injected into the empty channels as a reference. Sensor chip regeneration and desorption were achieved by injecting 10 mM Gly-HCl buffer (pH 2.5) before the next round of analyses. The experimental data were processed using ProteOn manager software (version 2.0). The binding curves were processed for the start injection alignment and baseline. The reference-subtracted sensorgram was then fitted to the curves labeling a homogeneous 1:1 model. Protein surface data were grouped to fit the association (Ka) and dissociation (Kd) rate constants. The symmetry dissociation constant (KD) for the peptide-TLR4/MD2 interaction was evaluated by using the following equation:


(3) KD=Kd/Ka

### 2.14 Western blot analysis

Whole protein in the serum was collected using a whole-protein extraction kit (KeyGEN Biotech, Nanjing, China). The concentration of protein was determined with a BCA protein assay kit (KeyGEN Biotech, Nanjing, China). Proteins were fractionated by 10% polyacrylamide gels and transferred to polyvinylidene difluoride membranes. After blocking with 5% nonfat dried milk for 2 h at room temperature, membranes were immunoblotted with primary antibodies against phospho-IKKα/β, nuclear factor kappa-light-chain-enhancer of activated B cells (NF-κB; p65), phospho-NF-κB (p-NF-κB; p-p65), inhibitor of κB (IκB)-α, phospho-IκB-α (p-IκB-α), and β-actin-specific monoclonal antibodies (Santa Cruz Biotechnology, CA, USA; 1:1000) overnight at 4°C. Afterwards, membranes were incubated with HRP-conjugated secondary antibody (Santa Cruz Biotechnology; 1:1000) for 1 h at room temperature. The density of the specific proteins was quantified using the ChemiDoc MP Imaging System (Bio-Rad, Hercules, CA, USA).

### 2.15 Statistical analysis

Statistical analysis was performed using GraphPad Prism v7.0. Statistical comparisons were performed using Student’s t test. All data are expressed as the means ± SEM from at least 3 independent experiments. Significance was claimed with P values ≤ 0.05. NS: P > 0.05, *: P ≤ 0.05, **: P ≤ 0.01, ***: P ≤ 0.001, ****: P ≤ 0.0001.

## 3 Results

### 3.1 Design of the peptides

**Figure 1:**
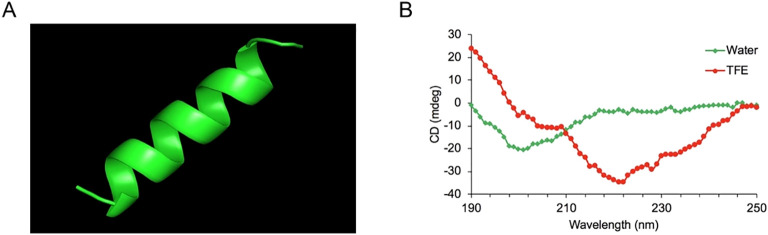
The overall structure of the hybrid peptide YTP. **(A)** The 3D structure of YTP generated through I-TASSER. **(B)** The CD spectra of YTP in 50% (v/v) TFE and water. The peptide was dissolved in sterile water or 50% TFE. The measurements were performed in the 190–250 nm (UV) range using a Jasco-810 spectropolarimeter at 25°C.

The sequences of the hybrid peptide YTP and its parental peptides YW12D and TP5 are shown in **Table 1**. The 3D structure of YTP was generated through I-TASSER and analyzed by PYMOL software (**Figure 1A**). In addition, structure determination by CD spectroscopy showed that YTP possesses an α-helical structure in 50% (v/v) TFE and a random coil structure in water (**Figure 1B**), which is consistent with the structure obtained through I-TASSER.

### 3.2 Cell proliferation assay

**Figure 2:**
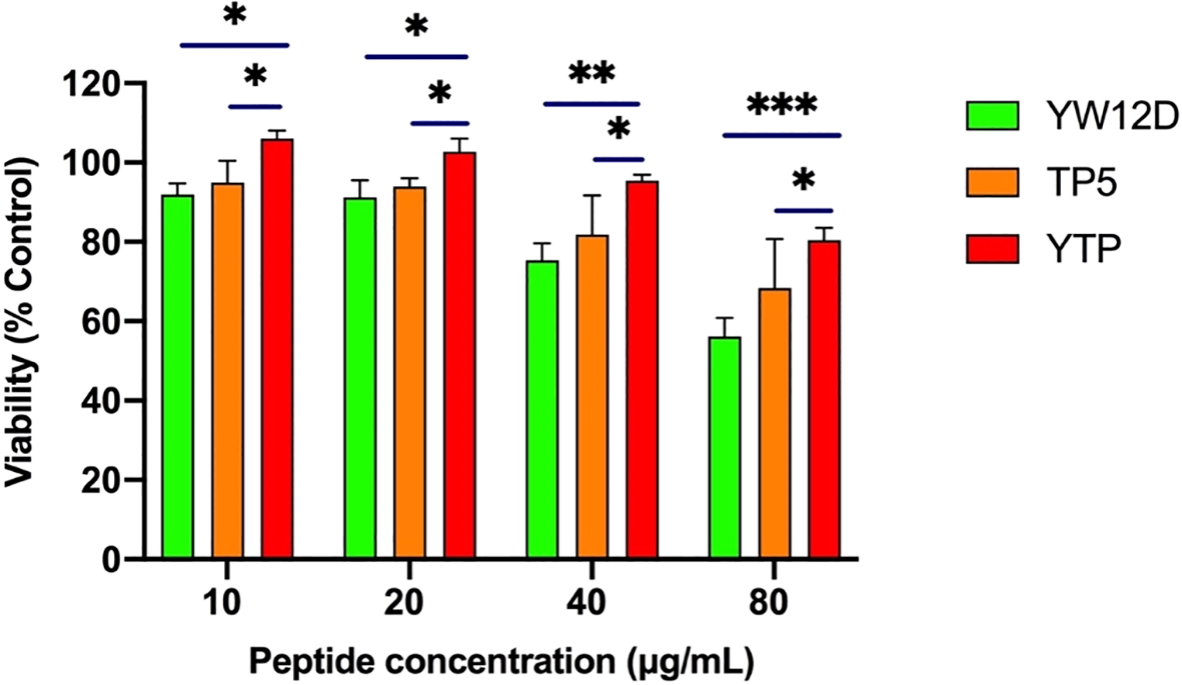
Cell viability of RAW264.7 macrophages. The data are presented as the mean ± SEM (n=8). NS: P > 0.05, *: P ≤ 0.05, **: P ≤ 0.01, and ***: P ≤ 0.001.

The cytotoxic activity of YTP against RAW264.7 macrophages was appraised by CCK-8 assay (**Figure 2**). The cytotoxicity of YTP was significantly lower than that of its parental peptides, YW12D and TP5, at all concentrations tested. In addition, YTP exhibited no significant cytotoxicity even at a high dose (80 µg/mL).

### 3.3 Ex vivo stability of YTP in plasma

**Figure 3:**
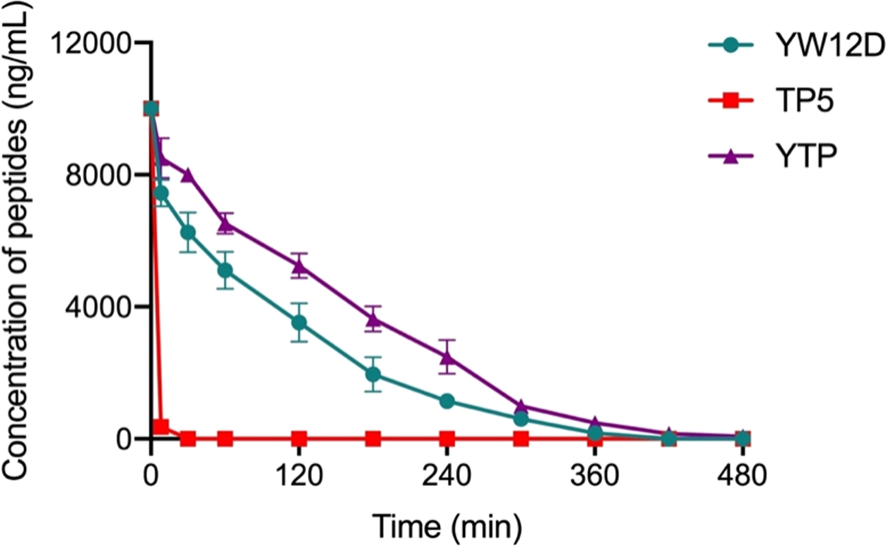
Concentrations of YW12D, TP5, and YTP in plasma over time. The concentration of each peptide in plasma *in vitro* at the selected time was quantified by HPLC. Data are given as the mean ± SEM (n = 5).

The plasma concentrations of YW12D, TP5, and YTP peptides over time are shown in **Figure 3**. TP5 showed a very short half-life, and its concentrations decreased nearly 95% after incubation in plasma for 5 min. The half-life of YTP in plasma was slightly more than 2 h, which was significantly longer than that of YW12D (P ≤ 0.01) and TP5 (P ≤ 0.0001) (**Figure 3**).

### 3.4 Effect of YTP on body weight and immune organs

**Figure 4:**
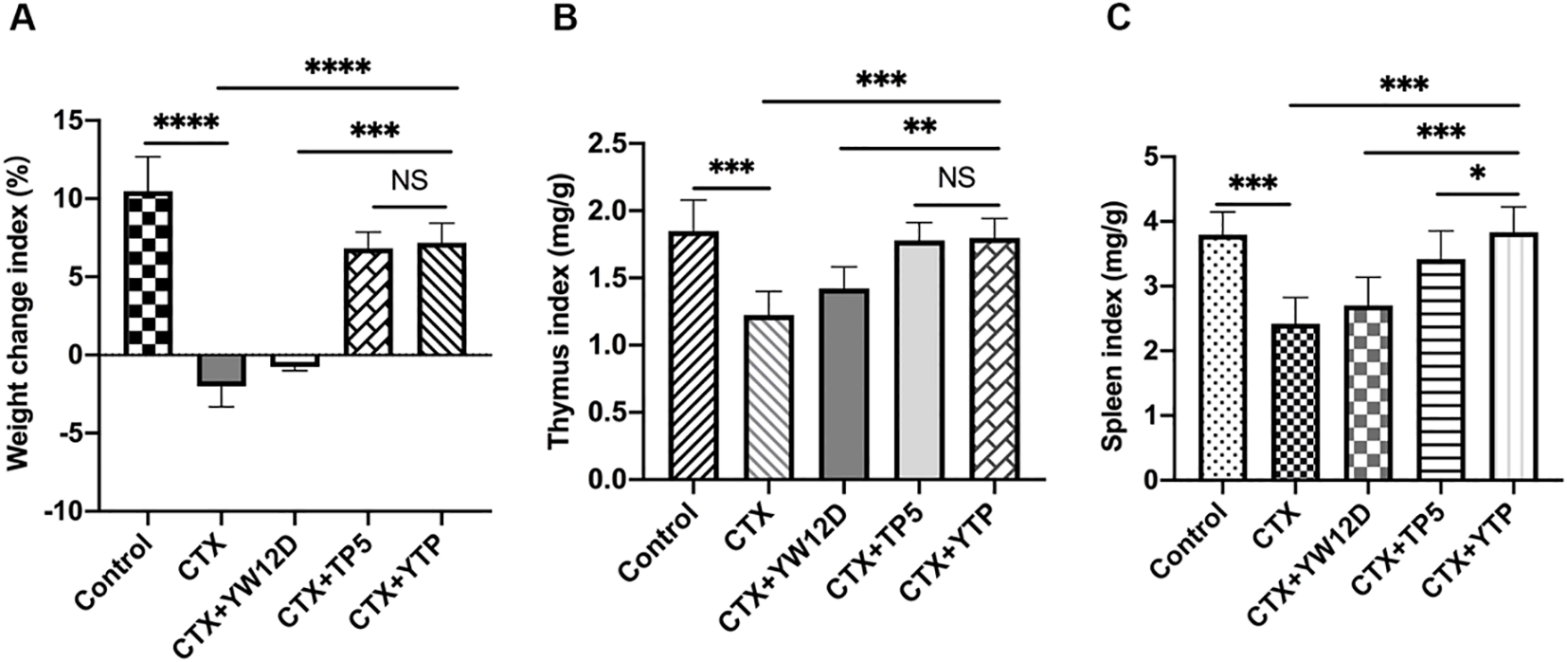
The effects of YW12D, TP5, and YTP on body weight **(A)**, thymus index **(B)** and spleen index **(C)**. The data are shown as the mean ± SEM (n=12). NS, P > 0.05; *, P ≤ 0.05; **, P ≤ 0.01; ***, P ≤ 0.001; and ****, P ≤ 0.0001.

As shown in **Figure 4**, the body weight of immunosuppressed mice in the CTX group were significantly reduced compared with those in the control group. In contrast to the CTX-induced group, mice in the CTX + YTP groups rapidly recovered their bodyweight. In addition, mouse body weight in the CTX + YTP group was significantly increased compared with that in the CTX + YW12D group (**Figure 4A**). As anticipated, the thymus (**Figure 4B**) and spleen (**Figure 4C**) indices were significantly decreased in the CTX-treated group. However, the administration of peptides remarkably improved the spleen and thymus indices, and the immune organ indices in the YTP-treated group were significantly increased compared with those in YW12D-treated and TP5-treated groups.

### 3.5 Effect of YTP on dendritic cell maturation

**Figure 5:**
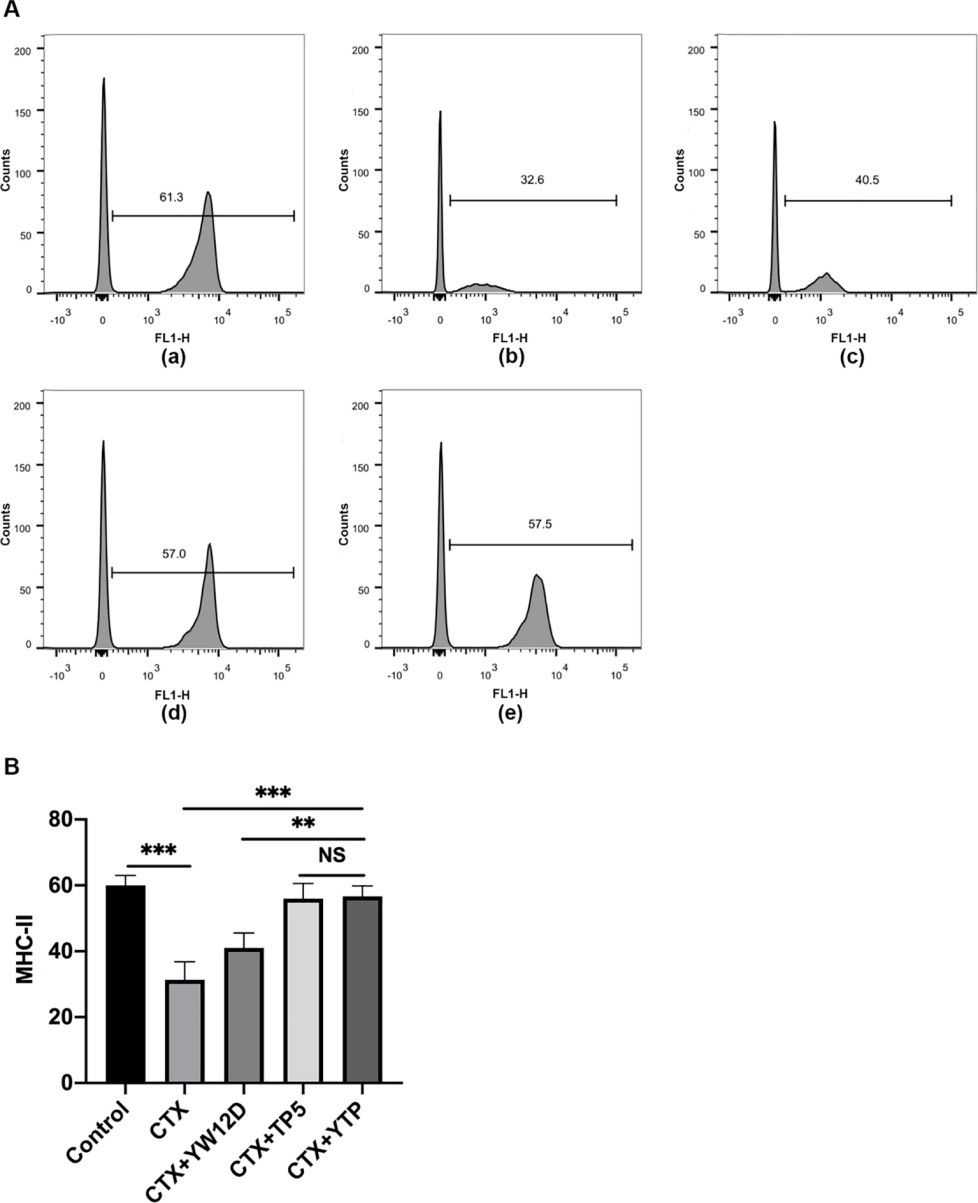
Effects of YW12D, TP5, and YTP on DC maturation. Mouse PBMCs were labeled with FITC-conjugated antibodies specific for MHC class II. **(A)** The surface expression of MHC-II was determined by flow cytometry. **(A-a)** Control, **(A-b)** cyclophosphamide (CTX), **(A-c)** CTX +YW12D, **(A-d)** CTX + TP5, **(A-e)** CTX + YTP. **(B)** The flow cytometry results were quantified and plotted. The data are presented as the mean ± SEM (n=12). NS, P > 0.05; **, P ≤ 0.01; ***, P ≤ 0.001.

To investigate the effect of YTP on serum DC maturation, the expression levels of DC phenotype factor (MHC-II) were determined by flow cytometry (**Figure 5**). The expression of MHC-II decreased significantly in the CTX group (**Figure 5A-b**) compared with that of the control group (**Figures 5A-a**, **B**). Treatment with YTP (**Figure 5A-e**) significantly increased the expression of MHC-II (**Figure 5B**). In addition, the expression of MHC-II in the YTP-treated group (**Figure 5A-e**) was significantly increased compared with that in the YW12D-treated group (**Figure 5A-c**) and similar to that in the TP5-treated group (**Figures 5A-d**, **B**).

### 3.6 Effects of YTP on T lymphocyte subpopulation

**Figure 6:**
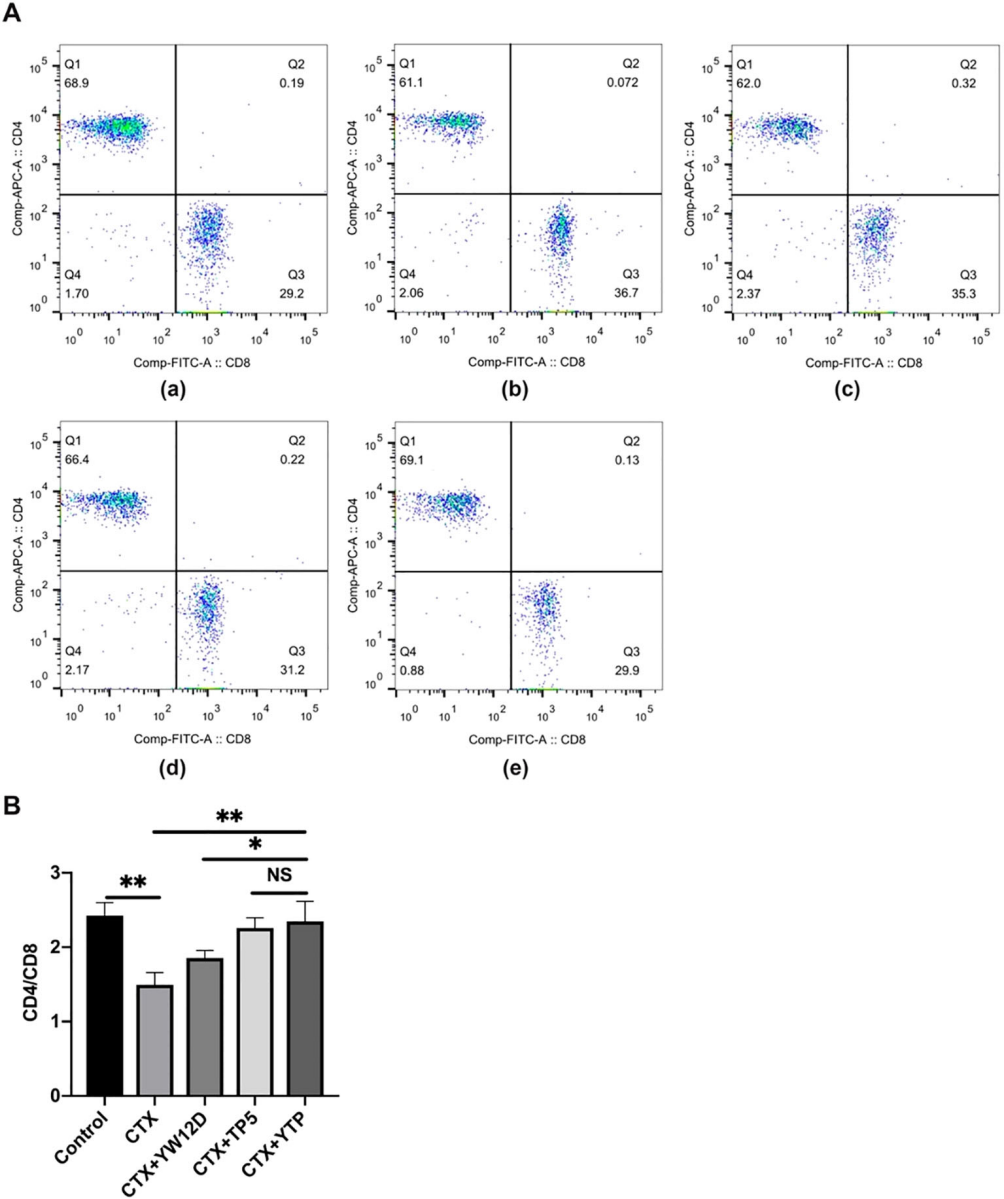
Effects of YW12D, TP5, and YTP on T lymphocyte subpopulations. **(A)** The percentage of different T cell subsets was analyzed by flow cytometry. (A-a) Control, (A-b) cyclophosphamide (CTX), (A-c) CTX + YW12D, (A-d) CTX + TP5, (A-e) CTX + YTP. Bivariate plots showed representative independent assessments, which were quantified and plotted as the CD4^+^:CD8^+^ ratio in **(B)**. The mean ± SEM (n = 12) is used to express the data. NS: P > 0.05, *: P ≤ 0.05, and **: P ≤ 0.01.

To characterize the immunomodulatory activities of YTP, the counts of CD4^+^ and CD8^+^ T lymphocytes in spleen were determined by flow cytometry. Compared with the control group (**Figure 6A-a**), CTX (**Figure 6A-b**) remarkably reduced the proportions of CD4^+^ and CD8^+^ T lymphocytes (**Figure 6B**), and treatment with peptides (**Figure 6A-e**) reversed this effect. In addition, the proportions of CD4^+^ and CD8^+^ T lymphocytes in the YTP-treated group (**Figure 6A-e**) was significantly higher than that in the YW12D-treated group (**Figure 6A-c**) and similar to that in the TP5-treated group (**Figures 6A-d**, **B**).

### 3.7 Effects of YTP on serum cytokines and immunoglobulin levels

**Figure 7:**
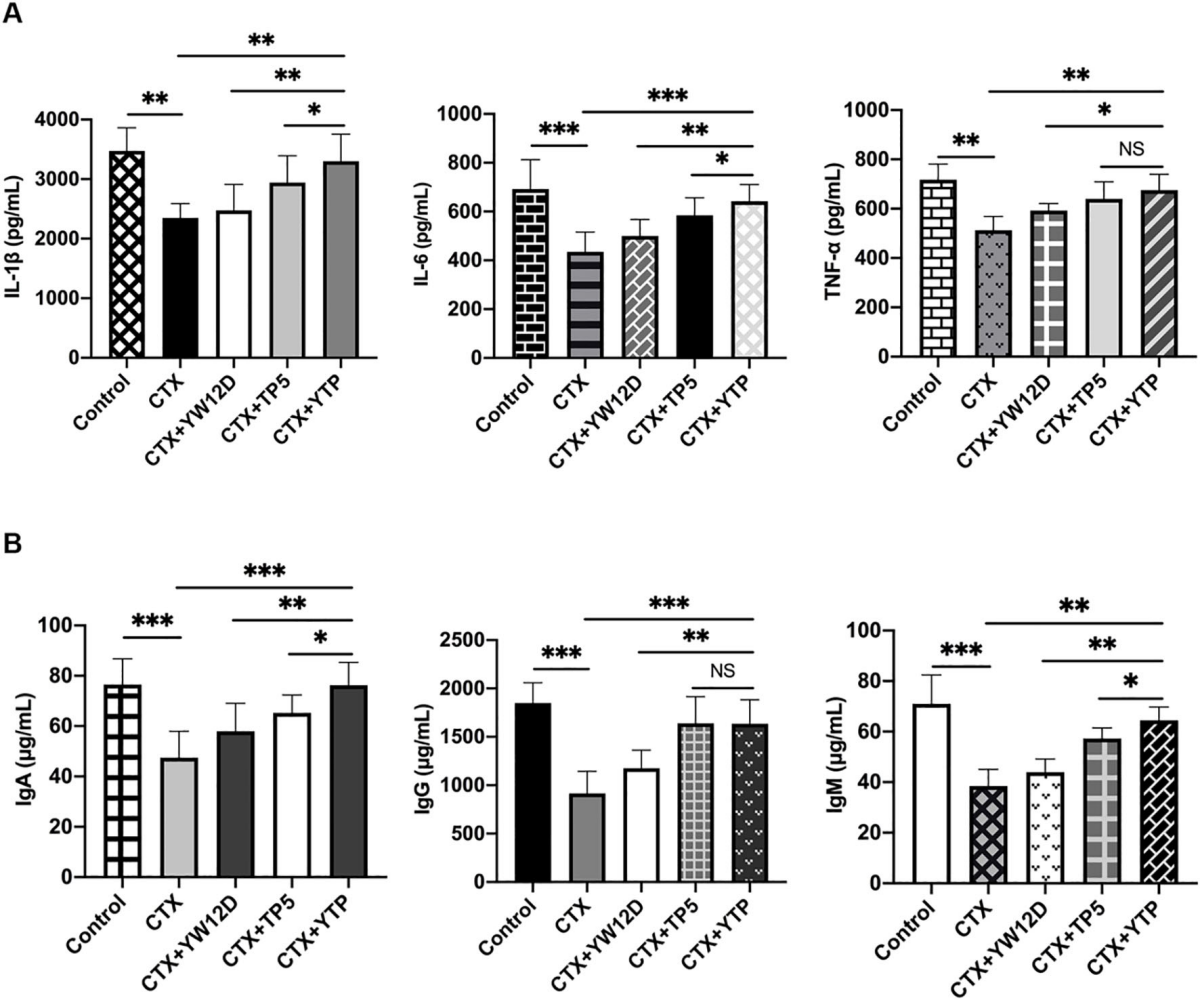
Effects of YW12D, TP5, and YTP on serum cytokine **(A)** and immunoglobulin **(B)** expression. Levels of IL-1β, IL-6, TNF-α, IgA, IgG, and IgM in the serum of mice were determined through ELISA. The data are presented as the mean ± SEM (n=12). NS, P > 0.05; *, P ≤ 0.05; **, P ≤ 0.01; and ***, P ≤ 0.001.

To investigate the immunomodulatory activity of YTP in CTX-treated mice, serum IL-1β, IL-6, and TNF-α levels were evaluated by ELISA (**Figure 7**). As shown in **Figure 7A**, the IL-1β, IL-6, and TNF-α levels in the CTX + TP5 and CTX +YTP groups were significantly increased compared with the CTX-induced group. In addition, mice in the CTX +YTP group exhibited markedly increased IL-1β, IL-6, and TNF-α expression compared with the CTX + YW12D and CTX + TP5 groups (**Figure 7A**).

Furthermore, CTX treatment resulted in significant reductions in IgA, IgG and IgM levels (**Figure 7B**). The administration of YTP caused a marked increase in all these immunoglobulin levels in the serum of mice. Notably, treatment with YTP resulted in higher IgA and IgM levels than in the CTX + YW12D and CTX + TP5 groups (**Figure 7B**).

### 3.8 YTP interacts directly with TLR2 and activates NF-кB signaling pathway

**Figure 8:**
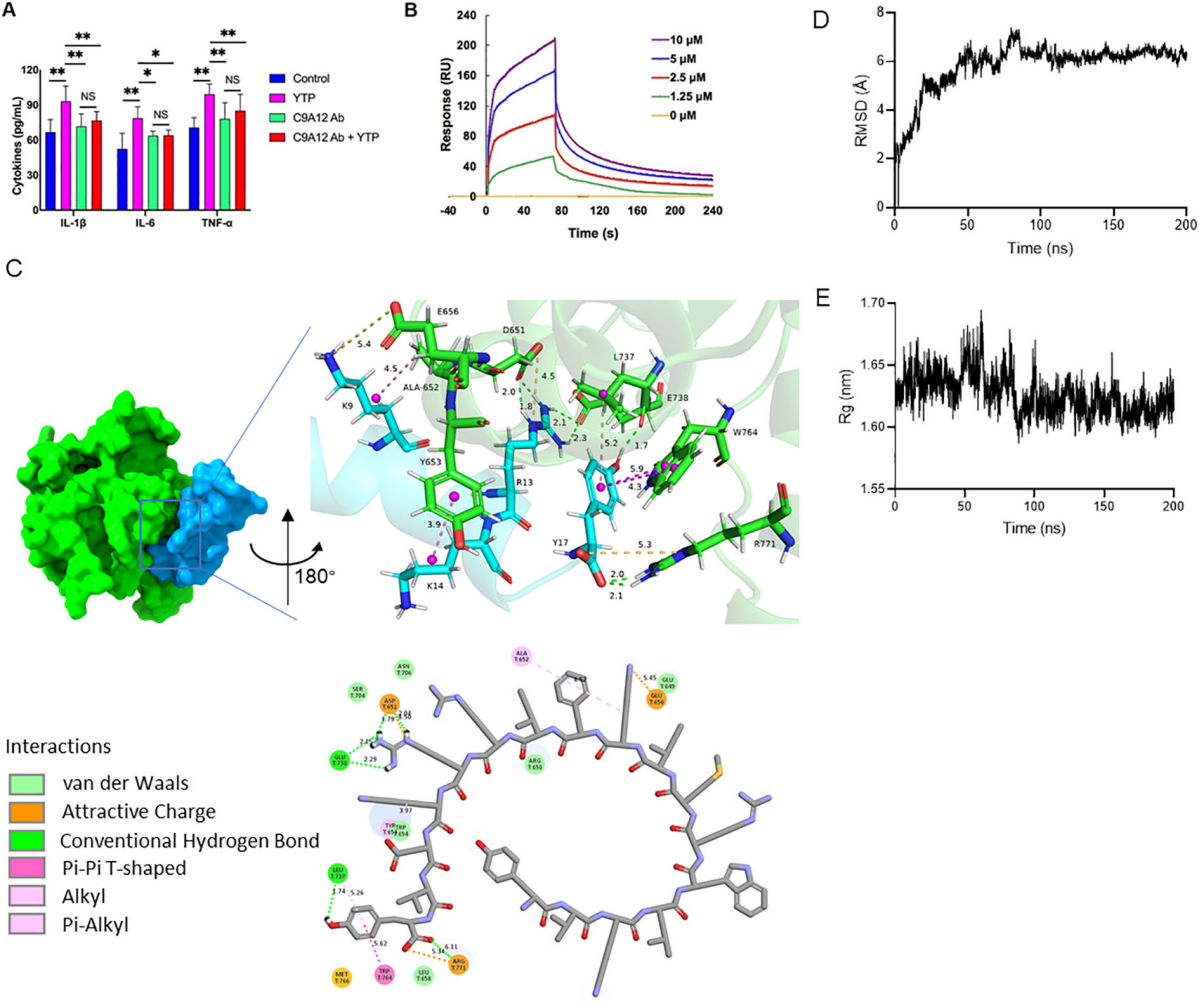
YTP interacts with TLR2 to activate TLR2 signaling. **(A)** RAW264.7 cells were treated with PBS or anti-mouse TLR2 mAb for 1 h with or without 10 μg/mL YTP peptide and further incubated for 24 h at 37°C. IL-1β, IL-6, and TNF-α levels in the culture supernatant were determined by ELISA. **(B)** SPR analysis of peptide YTP binding to TLR2. **(C)** The 3D and 2D docking models of TLR2 (PDB code: 1FYW) and YTP, along with the interaction results. **(D)** The root-mean-square-deviation (RMSD) value of TLR2 and YTP. **(E)** The radius of gyration (Rg) value of TLR2 and YTP. The data are presented as the mean ± SEM (n=5). NS, P > 0.05; *, P ≤ 0.05; and **, P ≤ 0.01.

To investigate the immunomodulatory mechanism of YTP, YTP binding to TLR2 was examined. RAW264.7 cells were incubated with PBS or TLR2 mAb (C9A12) for 1 h followed by treatment with or without 10 μg/mL YTP for 24 h. Then, IL-1β, IL-6, and TNF-α levels in the cell culture supernatant were quantified by ELISA (**Figure 8A**). YTP caused a significant increase in the production of IL-1β, IL-6, and TNF-α. Interestingly, pretreatment with TLR2 mAb significantly inhibited the TNF-α, IL-6, and IL-1β production induced by YTP, indicating that the YTP-TLR2 interaction is required for YTP immunomodulatory signaling activation.

Furthermore, to analyze the interaction of YTP and TLR2, an SPR assay was performed to evaluate the binding kinetics of ligand-receptor interactions in detail (**Figure 8B**). Five different concentrations of YTP (0, 1.25, 2.5, 5, and 10 μM) were passed over immobilized TLR2. As indicated in **Figure 7B**, YTP binding to the chip-bound protein exhibited a dose-dependent increase. The calculated Ka and Kd values for YTP binding to TLR2 were 4.31 × 10^6^ s^–1^ and 1.62 × 10^-2^ M^–1^s^–1^, respectively, and the KD value was 3.76 × 10^-3^ μM.

To better understand how YTP interacts with the TLR2 receptor, we used molecular docking and molecular dynamics simulations to analyze the binding pattern of the two molecules (**Figures 8C–E**). The complex trajectory analysis showed a stable RMSD value at 6 Å after approximately 100 ns (**Figure 8D**). The Rg value also stabilized at around 1.62 nm after 100 ns (**Figure 8E**). Key parameters of the interaction between YTP and TLR2 were analyzed, including electrostatic interactions (e.g., attractive charge), hydrogen bond, and hydrophobic interactions (e.g., Pi-Pi T-shaped, alkyl and Pi- alkyl) (**Figure 8C**; **Supplementary Table 1**). Fifteen pairs of interactions were formed between YTP and TLR2, in which the main amino acids involved in the interactions on YTP were Arg13 and Tyr17. Arg13 of YTP formed hydrogen bonds with G738 of TLR2 and formed both hydrogen bonds and an attractive charge with Asp651 of TLR2. As for Tyr17 of YTP, it formed a hydrogen bond and a hydrophobic bond with Leu737 of TLR2, two Pi-Pi T-shaped interactions with Trp764, and an attractive charge and two hydrogen bonds with Arg771, respectively. Both Arg13 and Tyr17 of YTP are derived from TP5, suggesting that the major contributor to the interaction between YTP and TLR2 is the parental peptide TP5. However, a few amino acids in YTP derived from YW12D (e.g., Lys9) also interacted with TLR2, which may have contributed to the slightly higher immunoreactivity of YTP than TP5 (**Figure 8C**, **Supplementary Table 1**).

**Figure 9:**
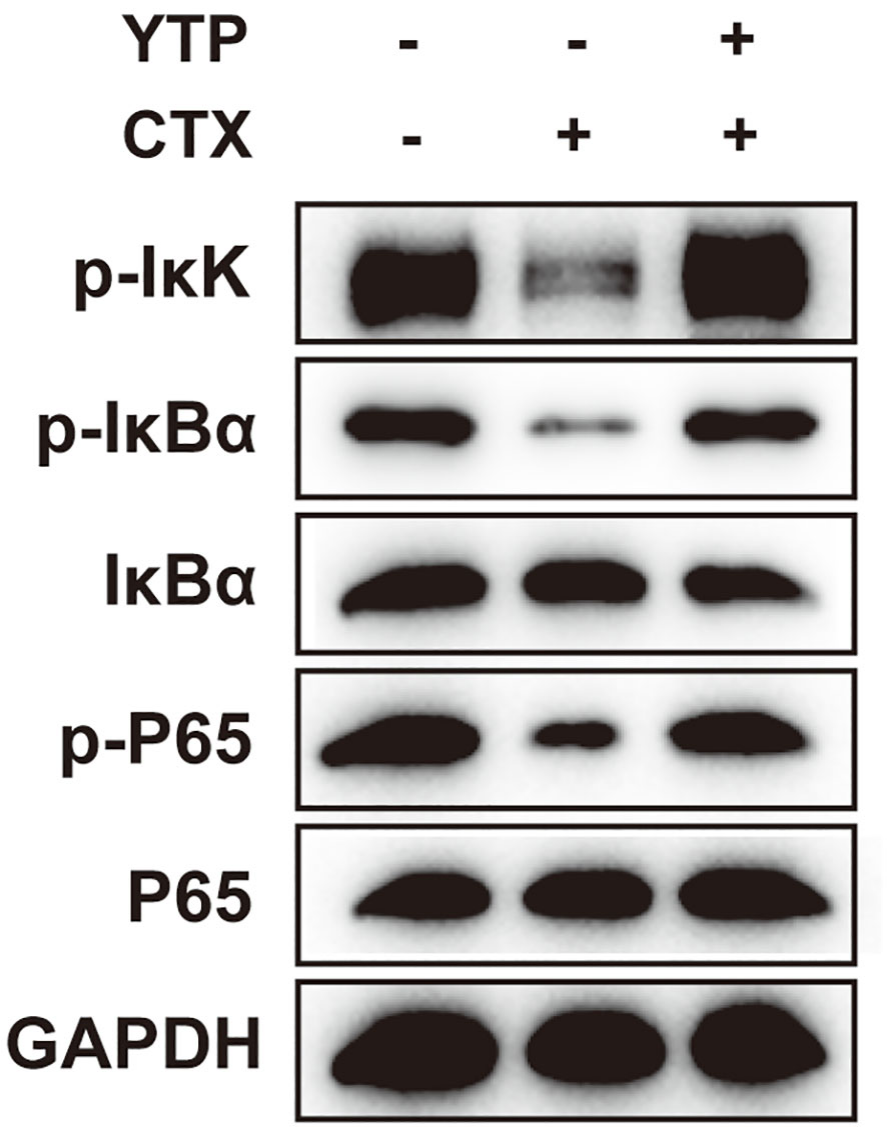
YTP activates the NF-кB signaling pathway. Phosphorylated and total protein levels of IkK, IκB-α and P65 from serum were measured by Western blot analysis.

Next, to investigate the mechanism underlying the immunomodulatory activity of YTP, we studied the downstream signaling pathway of TLR2 (**Figure 9**). IkK, IкB-α and P65 phosphorylation were reduced significantly after induction by CTX (**Figure 9**). However, levels of p-IkK, p-IкB-α and p-P65 were significantly elevated in mice treated with YTP after CTX induction (**Figure 9**). These data indicated that YTP exerts its immunomodulatory activity by activating the TLR2-NF-кB signaling pathway.

## 4 Discussion

Immunity can protect against disease by identifying and destroying harmful substances or toxins (1–3, 35, 36). As a state of temporary or permanent immune dysfunction, immunosuppression can make an organism more susceptible to infection, organ injury, and cancer due to damage to the immune system (4). It has been taken a long time to develop new immunomodulatory agents to prevent and treat immunosuppressive diseases, and progress has been slow (37). Hence, the design and development of new drugs with improved efficiency, safety and physiological stability are needed and complementary and alternative medicines are being sought. Recently, some peptides have been reported to have various effects and exhibit the potential to be used as treatments for a range of immunosuppression (10–13, 29, 38). However, the relatively limited immunomodulatory activity and poor physiological stability greatly hamper their clinical development (6, 12, 23–25, 39). Peptide hybridization is an effective approach for the design of novel peptides because hybrid peptides may have improved biological activity and stability compared to their parental peptides. Thus, the purpose of this study was to improve the immunomodulatory characteristics and increase the physiological stability of parental peptides by peptide hybridization.

TP5, one of the parental peptides of hybrid peptide YTP, plays a vitally important role in the process of immune enhancement (23, 24, 39). However, its bioavailability is greatly limited by its extremely short half-life *in vivo*, resulting in repeated injection and poor patient compliance (23, 24, 39). Given that the half-life of peptides significantly affects the dosage and therapy, it becomes an important task to prevent rapid degradation and prolong the half-life of such drugs. In this study, the half-life of YTP in plasma was prolonged to 120 min, which was significantly longer than that of YW12D or TP5 (less than 5 min).

CTX, as a typical immunosuppressant, can damage the structure of DNA, kill immune cells, interfere with the differentiation and proliferation of T and B cells, and decrease cellular and humoral immune responses (40, 41). Thus, the CTX-immunosuppressed murine model was used to examine the immunoregulatory effects of YTP in the present study. As expected, CTX stimulation of mice resulted in a significant decrease in the bodyweight and spleen and thymus index values. To furture understand the YTP immunoregulatory activities, several key cell types in the immune network were analyzed.

Dendritic cells (DCs) are a type of immune cell that play a critical role in the body’s immune system, particularly in linking the innate and adaptive immune responses. They are specialized in processing and presenting antigens to T cells, initiating an immune response (42, 43). Depending on environmental signals, DCs can modulate the immune response toward immunity or immune tolerance (42, 43). Mature DCs could upregulate MHC-II, display costimulatory molecules and produce cytokines, thereby playing an important role in immune induction and regulation (44). Therefore, DCs are potential targets for therapeutic intervention in immunosuppressive diseases (44). In the present study, the MHC-II expression level was tested to evaluate the effect of YTP on serum DC maturation. The MHC-II expression level was typically decreased in mice after CTX stimulation, whereas YTP treatment effectively upregulated the expression level, suggesting that YTP could efficiently enhance DC maturation in CTX-induced immunosuppressed mice. The T lymphocyte is a primary helper and effector cells in the adaptive immune response (45–47). Thus, it is important for the regulation of the immune response (48). When the immune system is suppressed, the organism is more susceptible to infection due to the decrease in the CD4^+^: CD8^+^ ratio (48). In this study, CTX remarkably reduced the proportions of CD4^+^ and CD8^+^ T lymphocytes, which was consistent with previous studies (11, 49). Treatments with peptides significantly increased the proportions, and the increasing level in the YTP-treated group was significantly higher than that in the YW12D-treated group. Thus, these results suggested that YTP improved immune function through regulating T lymphocyte subsets.

Immune enhancement of the host is related to the release of cytokines, such as TNF-α, IL-6, and IL-1β, which are involved in the preservation and restoration of homeostasis, activation and enhancement of immune properties, and the generation of other immunomodulatory cytokines (11, 50). In our study, the expression of immunoregulatory cytokines, such as TNF-α, IL-6, and IL-1β, was effectively increased by YW12D, TP5, and YTP. Notably, the increase in cytokine production by YTP is more potent than that by YW12D, which demonstrates that the immunomodulatory activities of the hybrid peptide YTP were stronger than those of the parental peptides.

Immunoglobulins potentially act as potentiators of the immune response in tissues via uptake of antigen to DCs (51). Furthermore, immunoglobulins can contribute directly to the immune response, including opsonizing antigens for destruction and fixation of complement and neutralization of toxins and viruses (52). This study showed that CTX stimulation significantly reduced the expression of immunoglobulins, such as IgA, IgG, and IgM. However, YTP treatment effectively attenuated the CTX-induced immunoglobulin decreases in the serum of mice. Notably, YTP promoted immunoglobulin production more potently than YW12D and TP5 at the same concentration.

Taken together, these results demonstrate that the newly designed hybrid peptide YTP has stronger immunomodulatory activity and physiological stability but lower cytotoxicity compared to its parental peptides. These findings strongly support the therapeutic potential of YTP against immunosuppression and hypoimmunity. To identify the underlying immunomodulatory mechanisms, a comprehensive and detailed analysis was conducted.

The immune system recognizes pathogen-associated molecular patterns and is involved in sensing endogenous danger signals through a series of pattern recognition receptors, such as Toll-like receptors (53, 54). As a critical signal transduction-associated membrane molecule, TLR2 is widely expressed on monocytes, mature macrophages and DCs, and mast cells (55, 56). Upon stimulation, TLR2 recruits the adapter molecules, TIRAP, IRAKs, and TRAF6 and then activates the IKK complex, leading to the activation of MAP kinases and NF-κB (57). These TLR2-mediated signaling pathways could regulate pro- and/or anti-inflammatory cytokine production, thereby triggering the activation of the immune response. Therefore, TLR2 ligands are potential molecules that can boost or block inherent immunoregulatory signal transduction and thereby act as pharmaceuticals to treat various autoimmune, inflammatory, immunosuppressive and malignant diseases. Given this background, we hypothesized whether the YTP peptide also exhibits immunoregulatory activities by activating the TLR2 signal transduction receptor. First, we performed ELISA assays to assess the interaction of YTP with the TLR2 receptor. The results showed that YTP robustly increased the expression levels of cytokines TNF-α, IL-6, and IL-1β in RAW264.7 cells. However, in the presence of TLR2 mAb, cytokine release in RAW264.7 cells exhibited a substantial decrease, indicating that YTP exerts the immunomodulatory effects through the interaction with the TLR2 signal transduction receptor. The SPR assay further confirmed that YTP can efficiently bind to the TLR2 protein in a dose-dependent manner. Molecular docking and molecular dynamics simulations were employed to investigate the detailed binding mode of YTP to TLR2. According to the results of the docking, the immunoreactivity of YTP mainly procide by the TP5, but at the same time YW12D contributed a little bit of additional intermolecular interactions. These results also explained the fact that YTP exhibits a superior immunoreactivity than TP5. Moreover, the results of molecular docking demonstrated the advantages of the hybrid peptide approch: it fully combines the strengths of the two parent peptides to achieve enhanced functionality.

A voluminous body of literature has revealed important roles for the NF-κB pathway downstream of TLR2 in regulating different aspects of immune functions (57). NF-κB-dependent signaling is a principal pathway that regulates cytokines, such as IL-1β, IL-6, and TNF-α, and cells that participate in the immunoregulating process (57). Given this background, we further assessed the changes in key factors involved in this pathway. The expression of major proteins in the NF-κB signaling pathway allowed elucidation of the immunomodulatory mechanism of YTP in CTX-induced immunosuppressed mice. Specifically, YTP effectively promoted the activation of TLR2 and NF-κB signaling by accelerating the phosphorylation of p-IkK, p-IкB-α and p-P65.

In this study, the newly designed peptide YTP exhibits significantly lower cytotoxicity, along with higher immunomodulatory activity and physiological stability, compared to its parental peptides. YTP effectively inhibits immunosuppression and weight loss, increases immune organ indices, promotes DC maturation, and increases cytokine and immunoglobulin levels. The immunomodulatory effect of YTP is associated with its activation of the TLR2-NF-кB signaling axis. Collectively, peptide hybridization is a promising approach for the design and development of new peptides with enhanced bioavailability and limited adverse effects. The immunoregulatory potential of YTP can be exploited in technological and clinical applications, such as healthcare formulas, or as therapeutic immunoregulatory drugs for humans.

## Data Availability

The original contributions presented in the study are included in the article/**Supplementary Material**. Further inquiries can be directed to the corresponding author/s.
