# Supplementary material for: Molecular hybridization modification improves the stability and immunomodulatory activity of TP5 peptide
Source: Front Immunol. 2024 Nov 11;15:1472839. doi: 10.3389/fimmu.2024.1472839 (PMC11586334; doi:10.3389/fimmu.2024.1472839)
Supplement: Supplementary file 1 [file Table1.docx]

Supplementary Material

Molecular hybridization modification improves the stability and immunomodulatory activity of TP5 peptide

Junyong Wang^1^, Xuelian Zhao^1^, Yuan Tang^1^, Zetao Ding^1^, Maierhaba Aihemaiti^1^, Dayong Si^1^, Rijun Zhang^1^, and Xubiao Wei^1,2^ *

^1^ State Key Laboratory of Animal Nutrition and Feeding, College of Animal Science and Technology, China Agricultural University, Beijing 100193, China;

^2^ Lead contact

*** Correspondence:**Xubiao Wei
weixubiao@cau.edu.cn (X.W.)

# Supplementary Tables

Table S1 Interactions between YTP and TLR2

| Name | Distance | Category | Types | From | From Chemistry | To | To Chemistry |
| --- | --- | --- | --- | --- | --- | --- | --- |
| T:ARG771:NE - Y:TYR17:OCT2 | 4.61017 | Electrostatic | Attractive Charge | T:ARG771:NE | Positive | Y:TYR17:OCT2 | Negative |
| Y:LYS9:NZ - T:GLU656:OE2 | 5.44566 | Electrostatic | Attractive Charge | Y:LYS9:NZ | Positive | T:GLU656:OE2 | Negative |
| Y:ARG13:NE - T:ASP651:OD2 | 4.50423 | Electrostatic | Attractive Charge | Y:ARG13:NE | Positive | T:ASP651:OD2 | Negative |
| T:ARG771:HH12 - Y:TYR17:OCT2 | 2.03558 | Hydrogen Bond | Conventional Hydrogen Bond | T:ARG771:HH12 | H-Donor | Y:TYR17:OCT2 | H-Acceptor |
| T:ARG771:HH22 - Y:TYR17:OCT2 | 2.09061 | Hydrogen Bond | Conventional Hydrogen Bond | T:ARG771:HH22 | H-Donor | Y:TYR17:OCT2 | H-Acceptor |
| Y:ARG13:HE - T:ASP651:OD1 | 2.04382 | Hydrogen Bond | Conventional Hydrogen Bond | Y:ARG13:HE | H-Donor | T:ASP651:OD1 | H-Acceptor |
| Y:ARG13:HH12 - T:GLU738:OE1 | 2.28716 | Hydrogen Bond | Conventional Hydrogen Bond | Y:ARG13:HH12 | H-Donor | T:GLU738:OE1 | H-Acceptor |
| Y:ARG13:HH21 - T:ASP651:OD1 | 1.79284 | Hydrogen Bond | Conventional Hydrogen Bond | Y:ARG13:HH21 | H-Donor | T:ASP651:OD1 | H-Acceptor |
| Y:ARG13:HH22 - T:GLU738:OE1 | 2.14994 | Hydrogen Bond | Conventional Hydrogen Bond | Y:ARG13:HH22 | H-Donor | T:GLU738:OE1 | H-Acceptor |
| Y:TYR17:HH - T:LEU737:O | 1.74345 | Hydrogen Bond | Conventional Hydrogen Bond | Y:TYR17:HH | H-Donor | T:LEU737:O | H-Acceptor |
| T:TRP764 - Y:TYR17 | 4.31314 | Hydrophobic | Pi-Pi T-shaped | T:TRP764 | Pi-Orbitals | Y:TYR17 | Pi-Orbitals |
| T:TRP764 - Y:TYR17 | 5.94682 | Hydrophobic | Pi-Pi T-shaped | T:TRP764 | Pi-Orbitals | Y:TYR17 | Pi-Orbitals |
| T:ALA652 - Y:LYS9 | 4.57217 | Hydrophobic | Alkyl | T:ALA652 | Alkyl | Y:LYS9 | Alkyl |
| T:TYR653 - Y:LYS14 | 3.97434 | Hydrophobic | Pi-Alkyl | T:TYR653 | Pi-Orbitals | Y:LYS14 | Alkyl |
| Y:TYR17 - T:LEU737 | 5.25816 | Hydrophobic | Pi-Alkyl | Y:TYR17 | Pi-Orbitals | T:LEU737 | Alkyl |
